# An Elementary Treatise on Human Anatomy

**Published:** 1861-01

**Authors:** 


ART. IV.—*An Elementary Treatise on Human Anatomy.* By JOSEPH LEIDY, M.D., Professor of Anatomy in the University of Pennsylvania, etc. etc. Illustrated by 392 Engravings. 8vo., pp. 663. J. B. Lippincott & Co.: Philadelphia, 1860.

DR. JOSEPH LEIDY, as an ardent and intelligent cultivator of science, has few equals As a teacher of anatomy in one of the great and leading schools of the country, he has earned an enviable reputation. As a writer, his language, if not always classical, is never obscure or enigmatical. In a word, the Professor is one of the wide-awake men of the day; one of those who, by their talents and indomitable industry, have made their "sign" for posterity.

Dr. Leidy, in the work before us, appears in a new attitude. Heretofore, his name occurred only, or chiefly, in connection with the transactions of the Academy of Natural Sciences of Philadelphia, or the publications of the Smithsonian Institute at Washington, the pages of which bear ample evidence of the powers of his prolific pen. The Treatise on

Human Anatomy, recently issued from the press of Messrs. Lippincott & Co., is the first contribution which he has made to medical literature, properly so called.

In attempting to form a correct estimate of the merits of this work, which, as conscientious and truth-seeking reviewers, is our ardent desire, we must bear in mind two important circumstances: first, the object which Dr. Leidy had in view in composing it; and, secondly, the position which he occupies before the public as a non-medical practitioner; or, in other words, the fact that he is essentially, if not exclusively, a naturalist and a teacher of anatomy.

Before we proceed to discuss these topics, a few words may be said in reference to the necessity for such a work at this particular juncture in the history of our literature. The author seems to have been impelled to its publication by a laudable desire to gratify his pupils with a suitable text-book; and the manner in which he has performed his task is, as will appear by-and-by, at least highly creditable. That the work was really needed is a question which the reader must endeavor to solve for himself. There is certainly no dearth of treatises, native or foreign, upon the subject now, whatever there might have been twenty-five years ago. We remember the time when the principal text-books on anatomy were the works of Wistar, Bell, and Fyfe; and with what pleasure we greeted, as we waxed a little older, the treatises of Horner and of Quain, the latter in particular, because of its lucid style and its many other intrinsic excellencies. The appearance of the admirable System of Human Anatomy of H. Cloquet, translated by Dr. Knox, of Edinburgh, and reissued at Boston in 1830; and the Manual of General, Descriptive, and Pathological Anatomy of J. F. Meckel, translated by the late Dr. A. Sidney Doane, of New York, in 1832, and published in three portly octavo volumes, afforded us ineffable delight, such as a man thoroughly imbued with a genuine love for the science can alone experience. We felt happier, and for many years drew daily a longer breath, as we reflected upon these wonderful treatises, which we regarded very much with the same feeling with which the miser contemplates his hoarded gold. As time rolled on, the stock was augmented by the magnificent volumes of Quain and Sharpey, brought out, by-the-way, in this country, by the author of the treatise under consideration; by Granville Sharp Pattison's edition of Cruveilhier's matchless volume, entitled Human Anatomy, the very beau-ideal and perfection of such a work; by Erasmus Wilson's excellent System of Human Anatomy, edited by Dr. Goddard; and, lastly, the productions of our countrymen, Samuel George Morton, better known for his labors in natural science and ethnology; of T. G. Richardson, now Professor of Anatomy in the University of Louisiana; and of Dr. Handy, of Baltimore, besides some manuals of practical anatomy, as those of Agnew and Hodges.

The mere enumeration of these works shows the astonishing progress made during the last quarter of a century in anatomical writings, and how much greater the opportunities of the student are at the present day for acquiring a complete knowledge of the structure of the human body than in former times. The profuse illustrations which accompany most of the recent treatises on the subject form one of the most striking features of the art of teaching of the present age. During our pupilage such facilities were enjoyed only by the favored few, in the works of J. Cloquet, Mascagni, Lizars, and a few other masters of the science.

The work of the Philadelphia professor is an elementary one. This is clearly indicated by its title; it does not profess to be an elaborate treatise, and hence it should not be judged by a comparison with such productions as those of Hippolitus Cloquet, Quain and Sharpey, Meckel and Cruveilhier; productions which have earned for their authors not only a national reputation, but an enviable immortality, as worthy disciples of Andrew Vesalius, and of the anatomical school of the nineteenth century. These great masters have all written for the future as well as the present; their object was not an ephemeral notoriety, but an enduring fame, founded upon patient and laborious research, and an exactitude of description which challenges alike our gratitude and our admiration. The treatise of Dr. Leidy has no such lofty pretensions; his aim has been to supply his pupils with a convenient and useful text-book during their attendance upon his prelections, and no one that has examined its pages will say that he has fallen short of his object. If clearness and accuracy of description, systematic arrangement, and condensation of matter are commendable qualities, we know of no work on anatomy, native or foreign, which possesses these qualities in a more eminent degree. Nothing, indeed, could be more admirable, and in this, we conceive, consists one of the great merits of the book. Originality is not to be expected in a treatise on descriptive anatomy; all that an author can hope to do is to present his topics in as lucid, concise, and graphic a style as possible, so that the student may have no difficulty in following the teacher in the lecture-room, or in tracing out the parts upon the dead subject by his own unaided efforts. If we have any fault to find with Dr. Leidy, it is that he writes too much like a naturalist, and not enough as a medical man. His book is too rigidly descriptive. It is too tightly bound with the hoops of special anatomy. It eschews physiology and topographical anatomy altogether, or so nearly as to amount virtually to the same thing. It does not even deem it worth while to give, at any length, the combined actions and relations of the muscles, so important in regard to fractures and dislocations, and it nowhere affords us a satisfactory view of the anatomy of the more important surgical regions. In these respects we consider the work defective, and we have little doubt that the learned author must by this time think so himself. We know, however, of no one more competent to supply these deficiencies than he.

The microscopical part of the work bears evidence of Dr. Leidy's personal researches; brevity and clearness are here, as everywhere else, characteristic features. He fully believes in the value of the microscope, but does not permit it to take precedence of the knife.

The work is printed on yellow tinted paper, and is illustrated by three hundred and ninety-two wood-cuts, executed in the highest style of the art, by Mr. August Wilhelm, of this city. About one hundred and twenty of the engravings are original; the remainder having been selected from various sources. As a specimen of art—including paper, typography, and engraving—it is one of the most beautiful productions that have ever emanated from the American press. It is admirably adapted to the wants of the pupil, and will, no doubt, be well received by the profession generally.

We notice, as one of the most agreeable features of this work, a substitution of English for Latin names, with their proper accentuation. We have long been in favor of such a change, and we congratulate Dr. Leidy that he has had the courage to shake off the fetters of the old nomenclature, so distasteful to the true scholar, and so embarrassing to the student. In a MS. work on anatomy, written nearly twenty years ago, we adopted this method, afterwards followed, at our suggestion, by Professor Richardson, in his excellent work on anatomy, published by Messrs. Lippincott & Co., in 1855. We trust that the example set by these able teachers may have the effect of turning the minds of the other lights of the profession into the same channel. The anatomical jargon of the schools is an abomination, opposed alike to sound science and to good taste.

In order, apparently, to atone for this innovation, Dr. Leidy has deemed it necessary to give, in the form of foot-notes, what he calls "a copious synonymy." We cannot say that we approve of this arrangement. It would have been much more convenient for the pupil if the synonymy had been included in brackets in the same line with the English term. Moreover, the synonymy is exceedingly overburdened, recapitulating as it does all the barbarous names that have been applied to anatomical objects since the days of Herophilus. The following may serve as a specimen: the author is speaking of the molar teeth :—

"Dentes molares; large molars; true molars; dentes multicuspidati; dentes maxillares; clavales, or gomphii; molæ; mylodontes; mylæri; momisci; cheek teeth; wall teeth; log teeth; grinding teeth."

We can hardly imagine that the distinguished author could desire the pupil to charge his memory with such a mass of rubbish, now only to be found in old, obsolete works on anatomy, which no physician of the present day has either the inclination or the leisure to study. A few of the more common synonyms would be quite sufficient in any modern work on the subject, and the space thus consumed in this otherwise excellent work might, it seems to us, have been more usefully appropriated to topographical anatomy and the conjoined action of the muscles.

